# Supplementary material for: Comprehensive patient-level classification and quantification of driver events in TCGA PanCanAtlas cohorts
Source: PLoS Genet. 2022 Jan 14;18(1):e1009996. doi: 10.1371/journal.pgen.1009996 (PMC8759692; doi:10.1371/journal.pgen.1009996)
Supplement: S4 Files — (ZIP) [file pgen.1009996.s004.zip › Aneuploidy/PANCAN GISTIC2/cumulative histograms/Distribution_stages_cohorts/2021_11_23_15_3_distribution_stages_TGCT.pdf]

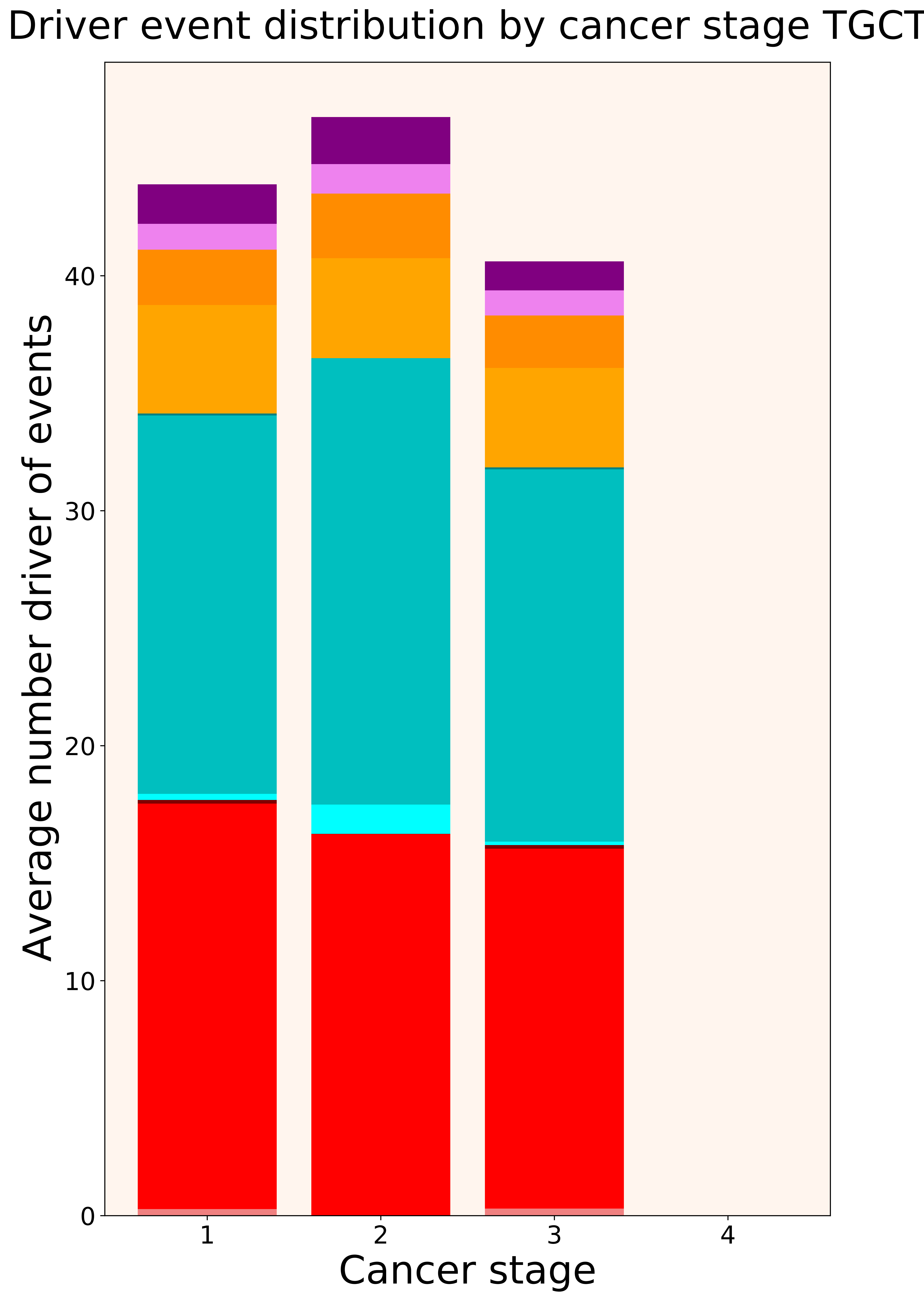
Driver event distribution by cancer stage TGCT
Average number driver of events
Cancer stage
0
10
20
30
40
1
2
3
4
